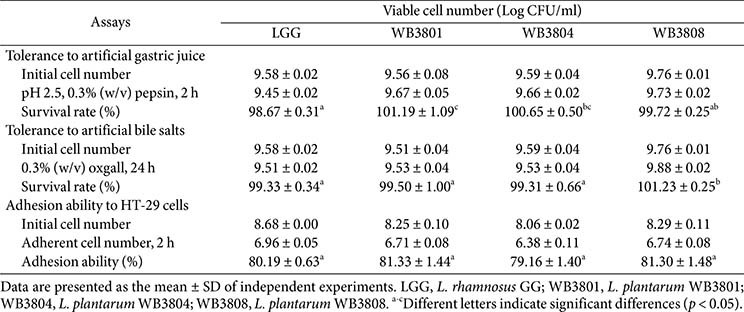# *Lactiplantibacillus plantarum* WB3801 and *Lactiplantibacillus plantarum* WB3808 Showed Antioxidant Effect and Anti-Apoptosis through Activation of the Keap1/Nrf2/HO-1 Pathway in H_2_O_2_-Induced HT-29 Cells

**DOI:** 10.4014/jmb.2508.08014

**Published:** 2025-10-15

**Authors:** Kyung-Min Park, Yu-Rim Lee, Na-Kyoung Lee, Hyun-Dong Paik

**Affiliations:** Department of Food Science and Biotechnology of Animal Resources, Konkuk University, Seoul 05029, Republic of Korea

**Keywords:** *Lactiplantibacillus plantarum*, oxidative stress, HT-29 cell, Nrf2/HO-1 pathway, antioxidant effect, apoptosis

## Abstract

Oxidative stress, primarily triggered by elevated levels of reactive oxygen species (ROS) contributes to intestinal epithelial injury and apoptosis, and is closely associated with gastrointestinal disorders. In this study, we evaluated the cytoprotective effects of three *Lactiplantibacillus plantarum* strains (WB3801, WB3804, and WB3808) isolated from Korean kimchi against H_2_O_2_-induced oxidative stress in HT-29 cells. The strains exhibited markedly strong acid and bile tolerance, as well as high adhesion to HT-29 cells, confirming their probiotic potential. These strains exhibited significantly improved cell viability under oxidative stress, reduced intracellular ROS levels, and preserved mitochondrial integrity. These strains enhanced cellular antioxidant defenses by activating the Keap1/Nrf2/HO-1 pathway, upregulating SOD1 and CAT, and demonstrating notable free radical scavenging activity in DPPH and ABTS assays. Flow cytometry and gene expression analyses revealed that WB3801 and WB3808 attenuated apoptosis by reducing the Bax/Bcl-2 ratio and suppressing the activation of caspase-9 and caspase-3. These findings suggest that WB3801 and WB3808 exert potent antioxidant and anti-apoptotic effects through both enzymatic defense and direct radical scavenging, ultimately protecting the intestinal epithelial cells from oxidative damage. The strong cytoprotective activity of these strains supports their potential use as functional probiotics for maintaining intestinal homeostasis and preventing oxidative stress–related intestinal disorders.

## Introduction

Oxidative stress refers to a state in which excessive generation of reactive oxygen species (ROS) exceeds the efficiency of their elimination mechanisms, such as H_2_O_2_ [1]. While produced as normal metabolites of aerobic respiration, they play vital roles in intracellular signaling and host defense, and their overproduction can overwhelm the cellular antioxidant defense system [2]. This imbalance leads to oxidative damage of essential biomolecules, causing DNA damage, protein modification, and lipid peroxidation, all of which contribute to chronic inflammation and tissue dysfunction [3]. Colorectal epithelial cells demonstrate increased sensitivity to oxidative stress due to prolonged exposure to microbial metabolites and dietary constituents within the intestinal lumen [4]. Patients with inflammatory bowel disease (IBD) show increased ROS production in the colonic mucosa, as detected using chemiluminescence assays. Notably, the exposure of HT-29 cells to H_2_O_2_ results in reduced ATP levels, indicating mitochondrial damage [5]. To mitigate these effects, cells rely on intrinsic antioxidant defense systems and DNA repair pathways.

The Kelch-like ECH-associated protein 1 (Keap1)/nuclear factor erythroid 2-related factor 2 (Nrf2)/heme oxygenase-1 (HO-1) pathway serves as a central modulator of the cellular antioxidant response to oxidative stress. Under homeostatic conditions, Nrf2 is retained in the cytoplasm by Keap1 and is quickly degraded. During oxidative stress, Nrf2 translocates into the nucleus, where it upregulates the expression of cytoprotective genes, including HO-1 and NAD(P)H Quinone Dehydrogenase 1 (NQO1) [6].

Apoptosis is a highly controlled mechanism of cell death necessary for maintaining physiological tissue balance and for the removal of genetically altered or damaged cells. Oxidative stress can initiate apoptosis through mitochondrial (intrinsic) pathway or through activation of death receptors (extrinsic pathway) [7]. Under conditions of cellular stress or damage, the permeability of mitochondrial membrane increases, causing cytochrome c to translocate into the cytoplasm. This triggers the activation of the initiators caspase-9 and caspase-3, consequently resulting in cell death [8].

Lactic acid bacteria (LAB), a major group of probiotic microorganisms, have gained attention owing to their antioxidant properties. LAB produce enzymes such as catalase (CAT), superoxide dismutase 1 (SOD1), and glutathione peroxidase (GPx), along with bioactive metabolites such as peptides, exopolysaccharides (EPS), and short-chain fatty acids [9]. These compounds neutralize ROS and modulate redox-sensitive signaling pathways. Wang *et al*. [10] demonstrated that *Lactiplantibacillus plantarum* ZLP001 enhances antioxidant defense, reduces lipid peroxidation, and maintains epithelial integrity in H_2_O_2_-treated intestinal porcine epithelial cells. Moreover, this strain modulates apoptosis by regulating the expression of key apoptosis-related proteins, including caspase, B-cell lymphoma 2 (Bcl-2), and Bcl-2 associated X (Bax).

Among the various health benefits of LAB, their antioxidant activity and ability to prevent cell death are particularly important for maintaining intestinal homeostasis. However, the mechanisms through which LAB alleviate oxidative stress and modulate cell death in human intestinal epithelial cells remain unclear. Therefore, this study was conducted to evaluate the cytoprotective effects of *L. plantarum* WB3801, *L. plantarum* WB3804, and *L. plantarum* WB3808 in H_2_O_2_-induced oxidative stress conditions in HT-29 cells, with a particular focus on the Keap1/Nrf2/HO-1 signaling pathway and the modulation of proteins related to apoptosis.

## Materials and Methods

### Sample Preparation

Bacterial strains WB3801, WB3804, and WB3808 were isolated from Korean cabbage kimchi. They were identified by the 16S rRNA gene sequencing using primers 27F and 1492R. As a reference strain, the commercial probiotic *Lacticaseibacillus rhamnosus* GG (LGG) was obtained from the Korean Collection for Type Cultures (KCTC, Republic of Korea). All the strains were preserved at –80°C in 20% (*v/v*) glycerol stocks.

For bacterial cultivation, the LAB strains were cultured in de Man, Rogosa, and Sharpe (MRS, Difco Laboratories, Inc., USA) at 37°C for 18 h. Following incubation, bacterial cells were collected by centrifugation at 13,500 ×*g* for 5min at 4°C using MICRO 17TR (Hanil Science Co., Republic of Korea), and the supernatant was removed. The pellet was rinsed twice with phosphate-buffered saline (PBS, HyClone Inc., USA). Subsequently, the bacterial suspensions were adjusted to 1 × 10^8^CFU/ml in Roswell Park Memorial Institute 1640 medium (RPMI, HyClone Inc.). This concentration is widely adopted in *in vitro* studies to ensure sufficient interaction between probiotic bacteria and epithelial cells within a limited exposure time and surface area [11].

### Cell Culture

Human colon adenocarcinoma HT-29 cells (KCLB 30038) were obtained from the Korean Cell Line Bank (Republic of Korea). The cells were cultured in RPMI containing 10% fetal bovine serum and 1% streptomycin/penicillin solution. Cells were maintained in a humidified incubator with 5% CO_2_ at 37°C. The culture medium was replaced at two-day intervals, and upon reaching approximately 80% confluence, the cells were subcultured.

### Survivability of Strains in Artificial Gastric Juice and Bile Salts

Gastric juice and bile salt tolerance were evaluated according to the method described by Guo *et al*. [12], with slight modifications. For acid tolerance, LAB strains were inoculated into MRS broth adjusted to pH 2.5 and supplemented with 0.3% (w/v) pepsin (Sigma-Aldrich, USA) and incubated at 37°C for 2 h. For bile salt tolerance, bacterial cells were harvested, washed, and resuspended in MRS broth containing 0.3% (w/v) Oxgall (Difco Laboratories Inc.), followed by incubation at 37°C for 24 h. Bacterial viability was determined by plating on MRS agar. The survival rate (%) under simulated gastrointestinal conditions was calculated using the following equation:

$$\text{Survival rate }\left(\%\right)=\left(\frac{\text{Cell number after incubation}\left(\text{Log CFU/ml}\right)}{\text{Initial cell number}\left(\text{Log CFU/ml}\right)}\right)\times 100$$

### Adhesion Ability

Adhesion of LAB strains to HT-29 cells was evaluated using the method described by Yang *et al*. [13]. LAB suspensions were subsequently added to the HT-29 confluent monolayers, followed by incubation at 37°C for 2 h. Non-adherent bacteria were removed by washing the wells three times with PBS. To collect the adherent bacteria, each well was treated with 0.1% (*v/v*) Triton X-100 (Sigma-Aldrich). The resulting lysates were serially diluted and plated onto MRS plates to enumerate the adherent bacteria. The adhesion (%) of HT-29 cells was calculated using the following formula:

$$\text{Adhesion ability }\left(\%\right)=\left(\frac{\text{Attached bacterial number}\left(\text{Log CFU/ml}\right)}{\text{Initial bacterial number}\left(\text{Log CFU/ml}\right)}\right)\times 100$$

### Cell Viability

Cell viability was assessed using the 3-(4,5-dimethylthiazol-2-yl)-2,5-diphenyltetrazolium bromide (MTT) assay (Sigma-Aldrich, USA) as previously described by Bock *et al*. [14]. HT-29 cells were seeded in 96-well plates at a density of 1 × 10^5^ cells/well and incubated for 24 h to allow for cell attachment. To determine the concentration of H_2_O_2_, cells were treated with varying concentrations of H_2_O_2_ (0.5, 0.75, 1.0, 1.25, 1.5, 1.75, and 2.0 mM) for 24 h, and viability was measured. Concentration 1.25 mM H_2_O_2_ was selected. After incubation, LAB strains were added for 2 h, followed by treatment with 1.25 mM H_2_O_2_ for an additional 22 h. Following treatment, the cells were washed twice with PBS, and MTT solution (5 mg/ml) was added to each well. After further incubation, the supernatant was removed, formazan crystals were solubilized in dimethyl sulfoxide, and absorbance was determined at 570 nm. The percentage of viable cells was calculated relative to the absorbance of the control group, as follows:

$$\text{Cell viability }\left(\%\right)=\left(\frac{{\text{OD}}_{\text{Sample}}}{{\text{OD}}_{\text{Control}}}\right)\times 100$$

### ROS Production

Intracellular ROS levels were assessed using a fluorescence-based method adapted from that reported by Agarwal *et al*. [15] with slight modifications. HT-29 cells were seeded into 12 well plates and cultured at 37°C. After 2 h, bacterial samples were added, and the cells were treated with 1.25 mM H_2_O_2_ for 18–24 h. Following incubation, the wells were washed with PBS to remove the residual medium. The cells were treated with 20 μM 2',7'-Dichlorodihydrofluorescein diacetate (DCFH-DA, Sigma-Aldrich) and incubated in the dark for 30 min without disturbance. Fluorescence images were visualized using a fluorescence microscope (Nikon Co. Ltd., Japan) and images were captured using a DS-Ri2 digital camera (Nikon Co. Ltd.).

### Cell Apoptosis Analysis

Apoptosis was quantified by flow cytometry using an FITC Annexin V/Dead Cell Apoptosis Kit (Thermo Fisher Scientific, USA). HT-29 cells were washed three times with PBS and centrifuged at 500 ×*g* for 5 min at 4°C. The cell pellets were resuspended in 100 μl of Annexin V binding buffer and subsequently incubated with 5 μl FITC-conjugated Annexin V in the dark at 25°C. Propidium iodide (PI) staining solution was prepared by diluting PI (1 mg/ml) in Annexing V binding buffer at a 1:9 ratio, and 100 μl was added to each sample. After 20 min, the samples were diluted with the binding buffer and analyzed using a flow cytometer [16].

### qRT-PCR

qRT-PCR was performed to evaluate the expression of antioxidant biomarkers (Nrf2, HO-1, NQO1, SOD1, and CAT) and apoptosis-related genes (Bax, Bcl-2, caspase-9, and caspase-3). HT-29 cells were seeded in 6-well plates (1 × 10^6^ cells/well) until a monolayer was formed. Following incubation with the LAB strains, oxidative stress was induced by treatment with H_2_O_2_ (1.25 mM) in all wells, except for the negative control. Total RNA was isolated using TRIzol reagent and cDNA was synthesized by reverse transcription using a cDNA synthesis kit (Bioline, UK). mRNA expression levels were evaluated by amplification using the PCR Master Mix (QuantStudio 1, Thermo Fisher Scientific, USA). The thermal cycling parameters were programmed following initial activation and denaturation at 95°C, followed by 50 cycles at 95°C for 15 sec, and 60°C for 60 sec [17]. Cycle threshold (Ct) values were normalized to β-actin, and relative gene expression was determined using the 2^-ΔΔCt^ method. The primer sequences used for qRT-PCR are listed in Table 1.

### Western Blot Analysis

HT-29 cells (1 × 10^6^ cells/well) were initially plated into 6-well plates and cultured for 3 days. The cells were pretreated with LAB strains for 2 h, followed by stimulation with 1.25 mM H_2_O_2_. Subsequently, lysis was performed using the Pro-Prep lysis buffer (iNtRON Biotechnology, Republic of Korea) containing protease and phosphatase inhibitors. Protein concentrations were determined, and 20 μg of each sample was resolved on SDS-PAGE using a 4% stacking gel and a 10% separating gel, followed by transfer onto polyvinylidene fluoride membranes. Membranes were blocked with 5% skim milk prepared in Tris-buffered saline containing 0.1%Tween 20 for 1 h at 25°C, followed by overnight incubation at 4°C with primary antibodies targeting Keap1, Nrf2, HO-1, SOD1, CAT, Bax, Bcl-2, caspase-9, and caspase-3 (Cell Signaling Technology, Inc., USA). After washing with TBST, membranes were incubated for 1 h at 25°C with secondary antibodies (1:10,000; Cell Signaling Technology Inc.). Protein bands were detected using an enhanced chemiluminescence detection kit (Advansta Inc., USA) on an X-ray film, and band intensity was quantified using ImageJ software (v. 1.54 m). Western blotting was conducted following a previously established method, with minor modifications [18].

### Antioxidant Assay

The antioxidant capacity of LAB strains was evaluated through 2,2-diphenyl-1-picrylhydrazyl (DPPH) and 2,2'-azino-bis-(3-ethylbenzothiazoline-6-sulfonic acid) (ABTS) radical scavenging assays, adapted from the method described by Lee *et al*. [19]. For the DPPH assay, a 0.1 mM DPPH solution was prepared in ethanol and mixed with the LAB suspension. The reaction mixture was incubated at 25°C for 30 min in the dark. Following incubation, the samples were centrifuged at 14,240 ×*g* for 1 min. Absorbance of the resulting supernatant was measured at 517 nm. DPPH radical scavenging activity was calculated using Eq. (1).

$$\text{Radical scavenging activity}\left(\%\right)=\left(1-\frac{{\text{OD}}_{\text{Sample}}}{{\text{OD}}_{\text{Control}}}\right)\times 100$$ (1)

For the ABTS assay, a radical stock solution was generated by reacting 14 mM ABTS with 5 mM potassium persulfate (1:1, *v/v*), followed by incubation in the dark at 25°C for 16–24 h. The solution was diluted with distilled water to achieve an absorbance of 0.70 ± 0.05 at 734 nm. Following this, 200 μl of the LAB suspension was added to 800 μl of the pre-diluted ABTS solution and incubated at 25°C for 15 min under dark conditions. The mixture was subsequently centrifuged at 14,240 ×*g* for 1 min, after which the absorbance of the supernatant was measured at 734 nm. The ABTS radical scavenging activity was calculated using Eq. (1).

### Statistical Analysis

All experiments were conducted in triplicate, and data were presented as mean values ± standard deviations (SD). Statistical analyses were carried out using SPSS software version 25.x. Differences among groups were evaluated using one-way ANOVA, followed by Duncan’s post-hoc test. Statistical significance was set at *p* < 0.05.

## Results

### Gastrointestinal Tolerance and Cell Adherence of LAB Strains

The probiotic characteristics of LAB strains isolated from kimchi were analyzed to assess their potential probiotic functionality (Table 2). The WB3801, WB3804, and WB3808 confirmed survival rates in both simulated gastric juice and artificial bile salts comparable to or exceeding that of LGG. In simulated gastric juice, WB3801 exhibited the highest acid tolerance, with a survival rate of 101.19%, which surpassed that of LGG (98.67%). WB3808 displayed the highest survival rate (101.23 %) among the artificial bile salts. These results highlighted the exceptional resilience of WB3801 and WB3808 to harsh gastrointestinal conditions.

The potential of the bacterial strains to attach to intestinal epithelial cells was evaluated. WB3801 showed the highest adhesion rate of 81.33%, while LGG showed slightly lower adhesion than WB3808, suggesting that the strains serve as probiotic strains with a high ability for intestinal cell attachment.

### Protective Effect of LAB Strains on H_2_O_2_-Induced Cytotoxicity

**Fig. 1:**
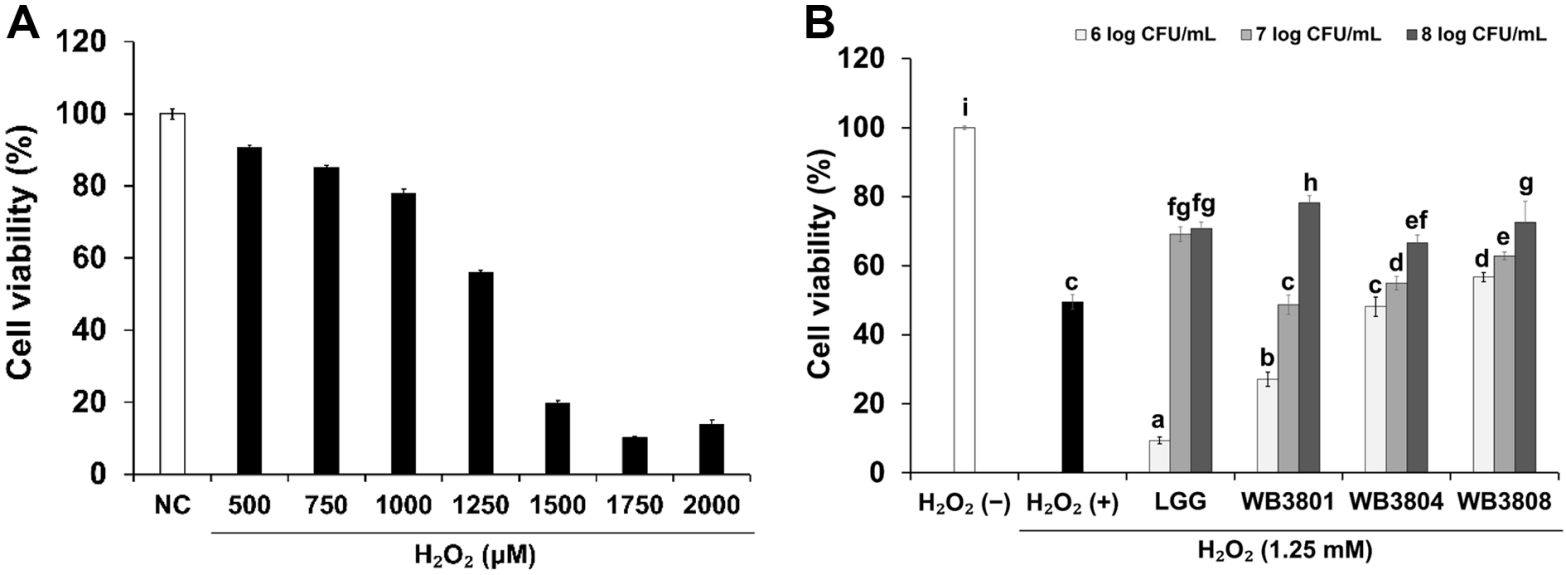
Effect of LAB strains on cell viability. (**A**) Effect of H_2_O_2_ on HT-29 cells. (**B**) Effect of different concentrations of LAB strains on H_2_O_2_-treated HT-29 cells. Data are presented as the mean ± SD of independent experiments. LGG, *L. rhamnosus* GG; WB3801, *L. plantarum* WB3801; WB3804, *L. plantarum* WB3804; WB3808, *L. plantarum* WB3808. ^a-i^Different letters indicate significant differences (*p* < 0.05).

To investigate the protective effects of the probiotics on H_2_O_2_-treated HT-29 cells, an MTT assay was performed (Fig. 1). In this study, the cytotoxic effects of different concentrations of H_2_O_2_ (0.5, 0.75, 1, 1.25, 1.5, 1.75, and 2 mM/ml) on HT-29 cells were tested. At 1.25 mM/ml H_2_O_2_, cell viability was 55.98%. Therefore, 1.25 mM H_2_O_2_ was selected as the concentration of H_2_O_2_ for the subsequent LAB strain experiments. As shown in Fig. 1B, Among the tested concentrations (6, 7, and 8 log CFU/ml), 8 log CFU/ml exhibited the most pronounced protective effect on cell viability. Therefore, this concentration was selected for subsequent experiments to ensure optimal probiotic cell interaction under oxidative stress conditions. Exposure to H_2_O_2_ (1.25 mM) significantly reduced cell viability to 49.51%. Otherwise, treatment with LGG, WB3801, or WB3808 attenuated cell death by more than 70%. WB3801 increased cell viability compared to H_2_O_2_-treated group.

### Reducing Effect Against H_2_O_2_-Induced ROS by LAB Strains

**Fig. 2:**
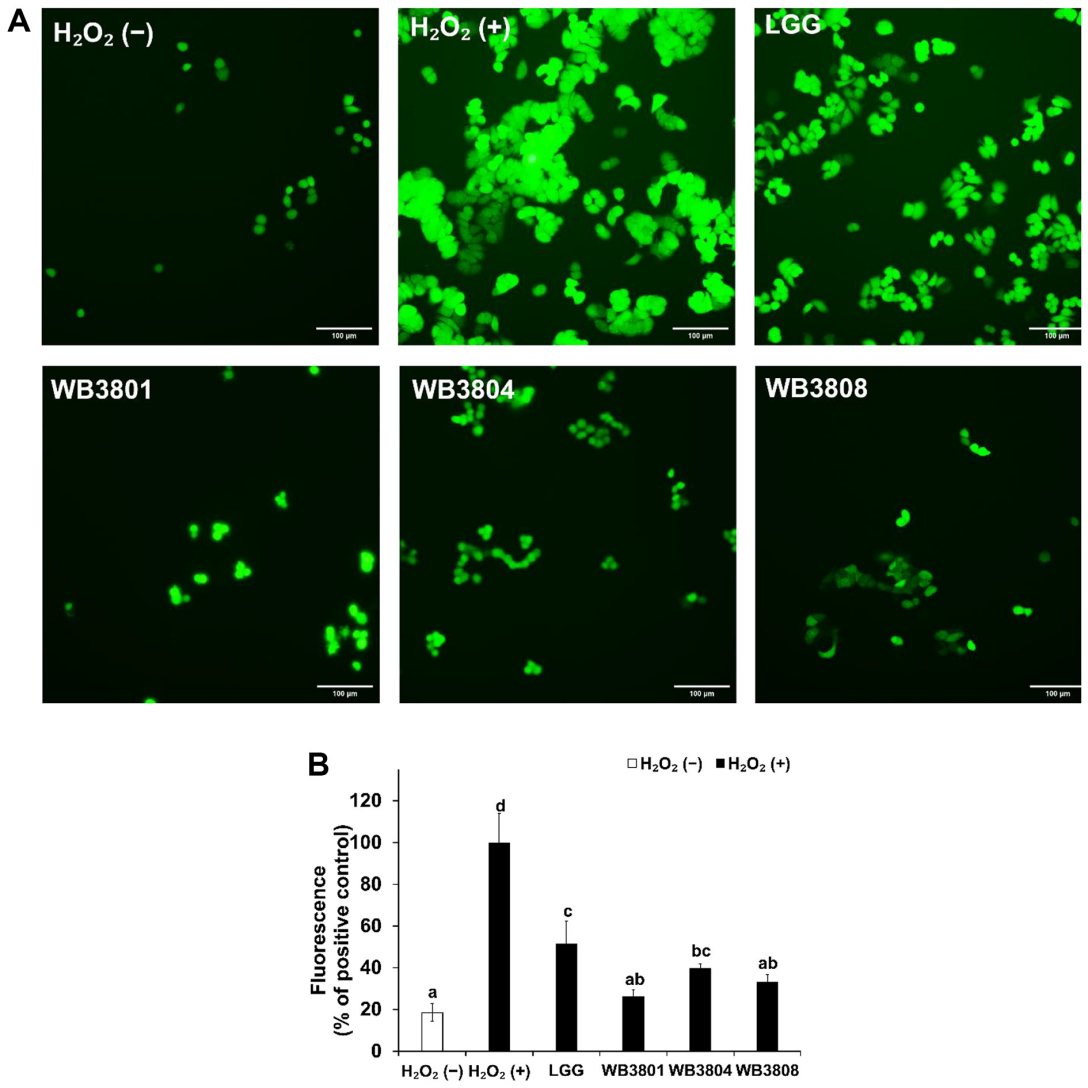
Effect of LAB strains on reactive oxygen species (ROS) generation in HT-29 cells. (**A**) DCFH-DA-stained HT-29 cells using fluorescence microscopy. (**B**) Fluorescence mean intensity. Data are presented as the mean ± SD of independent experiments. LGG, *L. rhamnosus* GG; WB3801, *L. plantarum* WB3801; WB3804, *L. plantarum* WB3804; WB3808, *L. plantarum* WB3808. ^a-d^Different letters indicate significant differences (*p* < 0.05).

DCFH-DA was used to examine ROS production in H_2_O_2_-induced HT-29 cells. As shown in Fig. 2, the exposure of cells to H_2_O_2_ increasingly promoted ROS generation. All groups treated with probiotics showed a significant reduction in ROS production compared with the group exposed to H_2_O_2_ alone. Among these, WB3801 showed the most prominent antioxidant effect (26.19%), with ROS levels reduced to values similar to those observed in the untreated group.

### Attenuation of H_2_O_2_-Induced Apoptosis in HT-29 Cells by LAB Strains

**Fig. 3:**
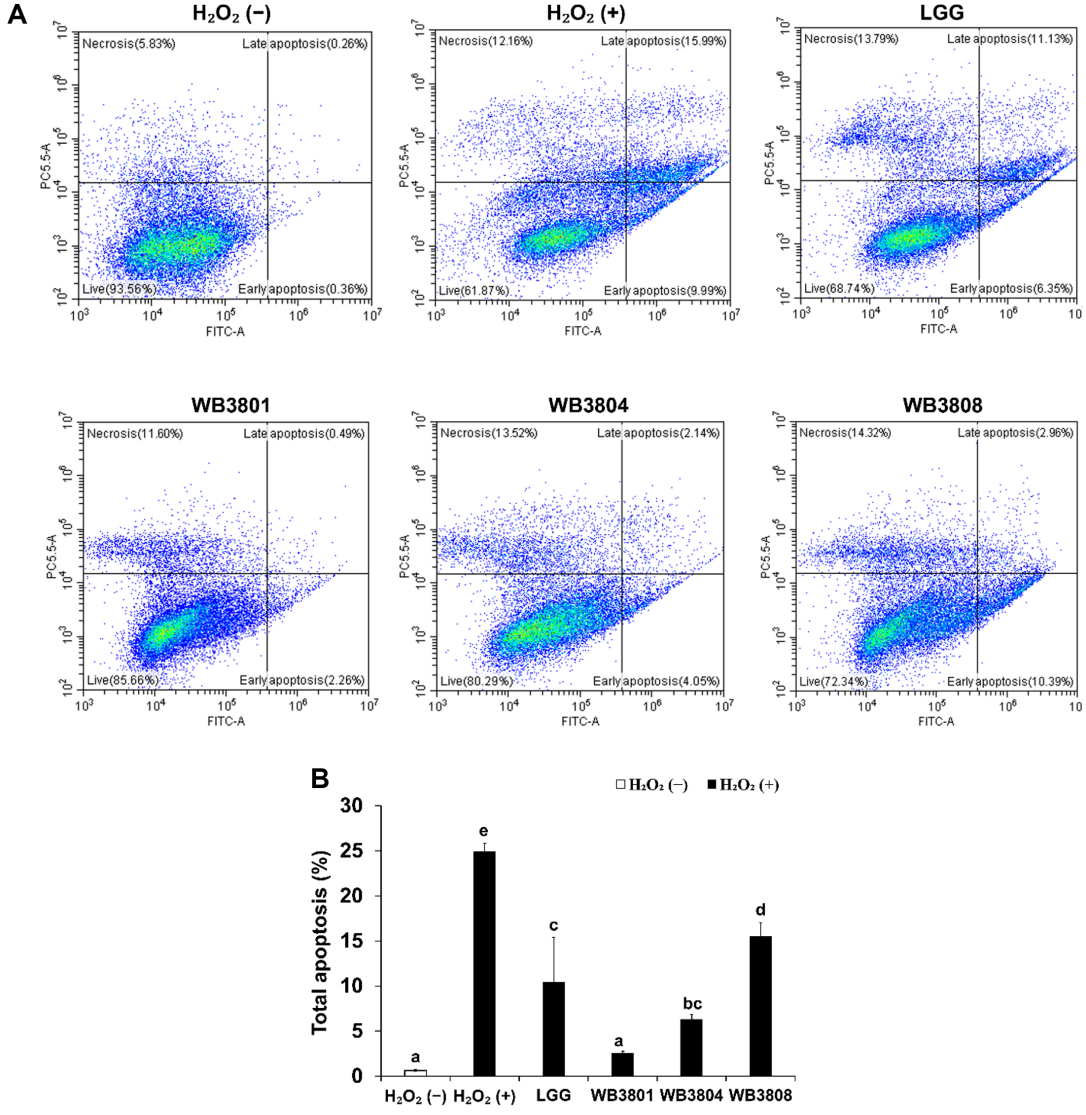
Apoptosis protection effects of LAB on H_2_O_2_-treated HT-29 cells. (**A**) Pseudocolor plots of PI and annexin V. (**B**) Total apoptosis (early and late apoptosis). Data are presented as the mean ± SD of independent experiments. LGG, *L. rhamnosus* GG; WB3801, *L. plantarum* WB3801; WB3804, *L. plantarum* WB3804; WB3808, *L. plantarum* WB3808. ^a-d^Different letters indicate significant differences (*p* < 0.05).

To examine the protective effects of the LAB strains against apoptosis, Annexin V-FITC/PI staining was conducted, followed by flow cytometry. As shown in Fig. 3, among the probiotic-treated groups, LGG attenuated apoptosis and reduced the total apoptotic rate to 10.45%. WB3804 and WB3808 effectively reduced apoptosis compared to the positive control. Notably, WB3801 exhibited the strongest anti-apoptotic effect, with total apoptosis suppressed to 2.51%, which approached that of the negative control.

### Upregulation of Antioxidant Genes and Downregulation Apoptosis Markers by LAB Strains

**Fig. 4:**
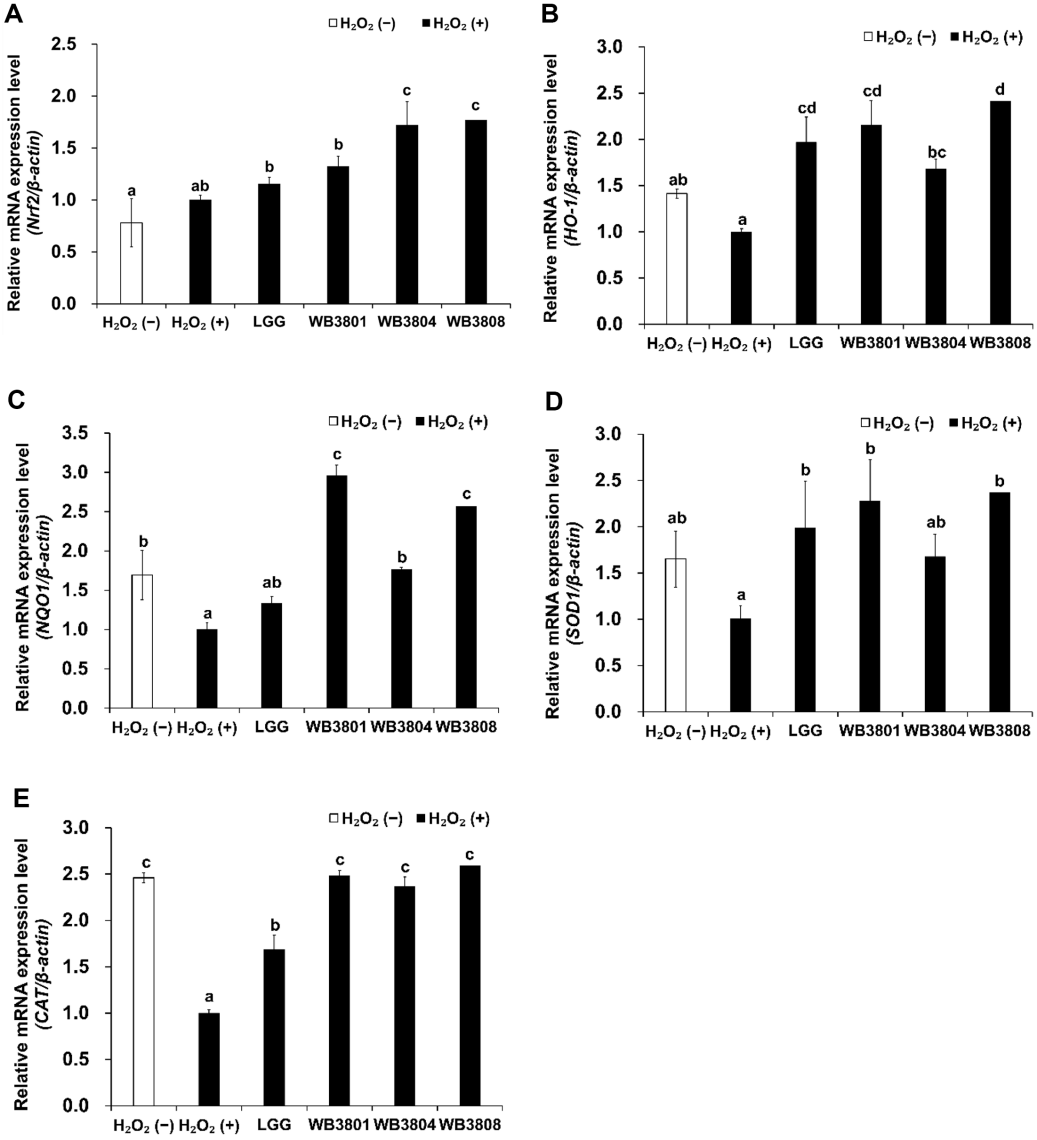
Antioxidant effects of LAB strains on H_2_O_2_-induced HT-29 cells. Expression of (**A**) Nrf2, (**B**) HO-1, (**C**) NQO1, (**D**) SOD1, and (**E**) CAT mRNA. Data are presented as the mean ± SD of independent experiments. LGG, *L. rhamnosus* GG; WB3801, *L. plantarum* WB3801; WB3804, *L. plantarum* WB3804; WB3808, *L. plantarum* WB3808. ^a-d^Different letters indicate significant differences (*p* < 0.05).

The relative mRNA gene expression levels of Nrf2, HO-1, NQO1, SOD1, and CAT, which indicate antioxidant effects, were significantly modulated by H_2_O_2_ and probiotic treatments (Fig. 4). In the non-treatment group, the expression of these factors was higher than that in the H_2_O_2_-treated group. Treatment with the probiotic strains markedly increased the mRNA gene expression levels of these factors compared to those in the H_2_O_2_ (−) group, with significantly higher levels compared to those in the H_2_O_2_ (+) group (*p* < 0.05). The three strains exhibited generally superior effects compared with LGG across all antioxidant-related markers. WB3801 induced over a twofold upregulation in the expression of all examined genes relative to the positive control, with the exception of Nrf2.

**Fig. 5:**
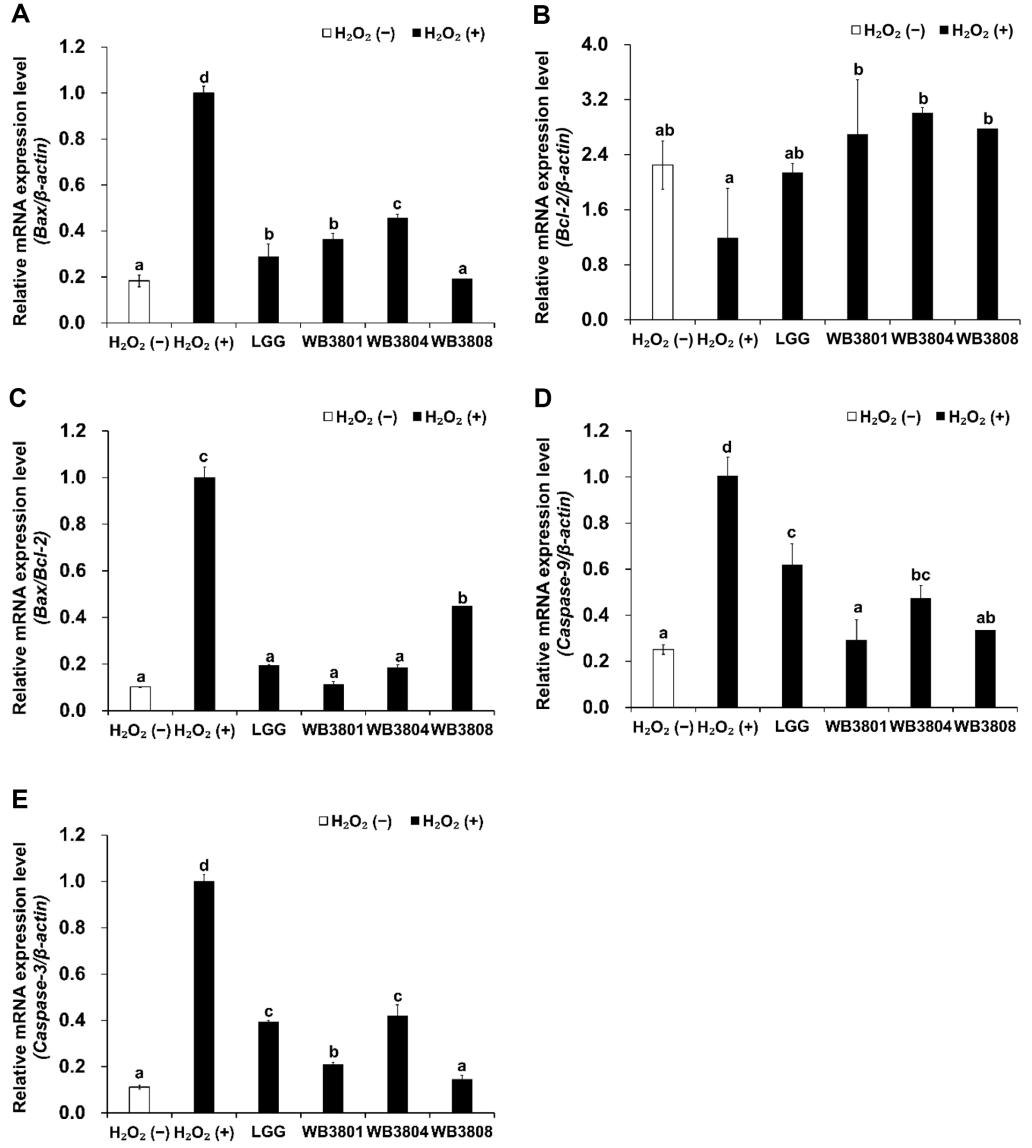
Downregulatory effect of LAB strains on the expression of apoptosis proteins in H_2_O_2_-induced HT-29 cells. mRNA levels of cytokines levels of (**A**) Bax, (**B**) Bcl-2, (**C**) Bax/Bcl-2, (**D**) caspase-9 and (**E**) caspase-3 using mRNA. Data are presented as the mean ± SD of independent experiments. LGG, *L. rhamnosus* GG; WB3801, *L. plantarum* WB3801; WB3804, *L. plantarum* WB3804; WB3808, *L. plantarum* WB3808. ^a-d^Different letters indicate significant differences (*p* < 0.05).

As shown in Fig. 5, treatment with the probiotic strains WB3801, WB3804, and WB3808 markedly reduced the mRNA expression levels of Bax, caspase-3, and caspase-9 compared with the H_2_O_2_ (+) group (*p* < 0.001), whereas all strains showed higher Bcl-2 expression levels than the H_2_O_2_ (+) group. The suppression of caspase-3, caspase-9, and Bax expression by WB3801 and WB3808 significantly downregulated than that by LGG. Regarding the expression of Bax/Bcl-2 ratio, a predictive marker of apoptotic response, the reduction of WB3801 exhibited the most pronounced suppression.

### Activation of Keap1/Nrf2/HO-1 Pathway and Suppression of Apoptosis Pathway by LAB Strains

**Fig. 6:**
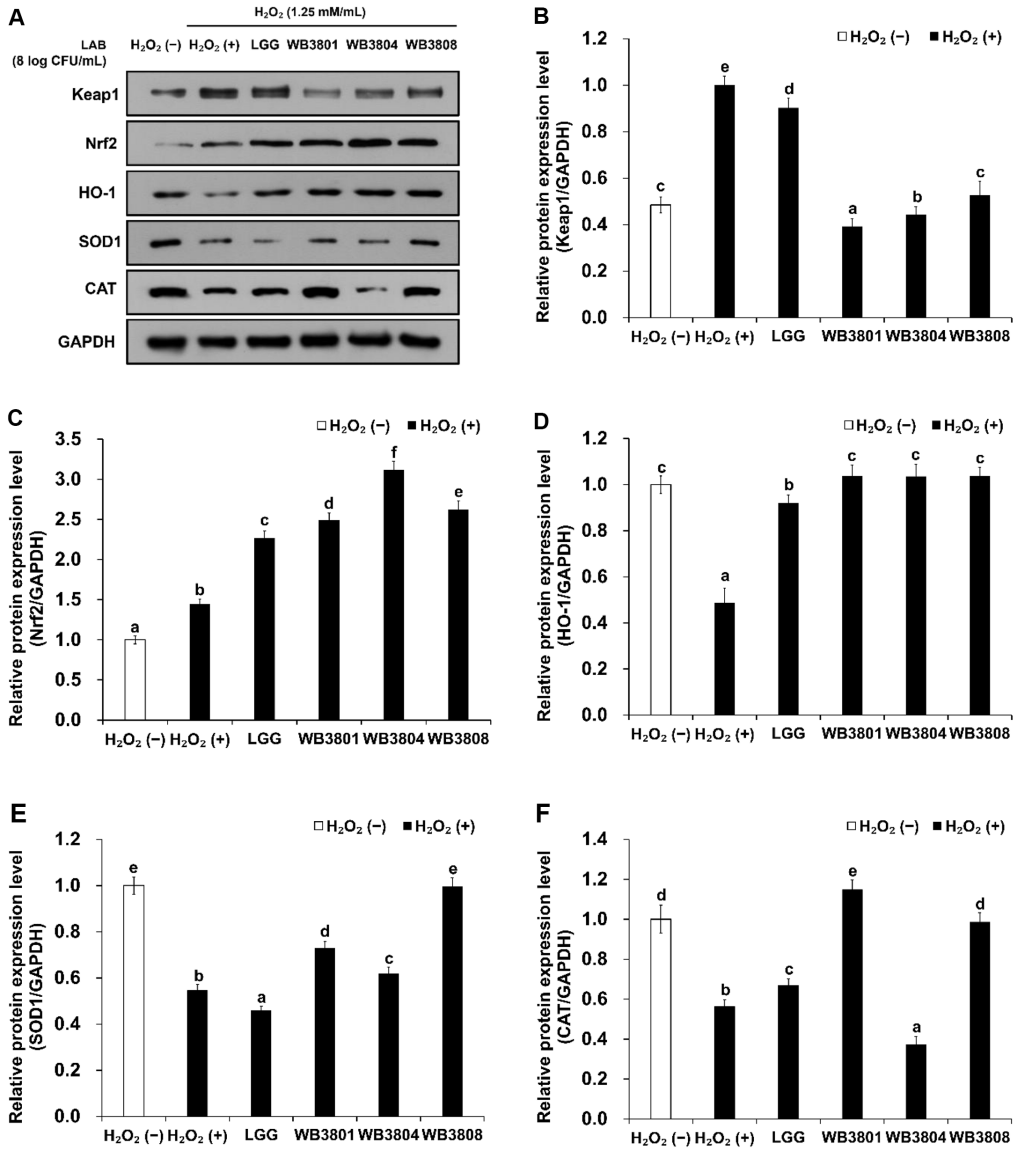
Effect of LAB strains on antioxidant pathway activation in H_2_O_2_-induced HT-29 cells. (**A**) Western blot analysis of Keap1/Nrf2/HO-1 pathway and antioxidant enzymes, (**B**) Keap1, (**C**) Nrf2, (**D**) HO-1, (**E**) SOD1, and (**F**) CAT. Data presented as the mean ± SD of independent experiments. LGG, *L. rhamnosus* GG; WB3801, *L. plantarum* WB3801; WB3804, *L. plantarum* WB3804; WB3808, *L. plantarum* WB3808. ^a-d^Different letters indicate significant differences (*p* < 0.05).

To assess the antioxidant effects of the tested strains, the expression levels of proteins associated with Keap1/Nrf2/HO-1 pathway were analyzed in H_2_O_2_-stimulated cells. As shown in Fig. 6, H_2_O_2_ treatment significantly increased Keap1 expression and decreased HO-1, SOD1, and CAT levels, indicating oxidative damage, compared to the untreated control. Keap1 expression was markedly downregulated in the WB3801 and WB3804 groups, whereas HO-1 expression was increasingly upregulated in all strain-treated groups compared to the LGG group. WB3808 showed the highest recovery of SOD1 expression, whereas WB3801 had the strongest effect on CAT upregulation. WB3804 cells showed the most pronounced induction of Nrf2. These results suggest that all WB strains activate the Keap1/Nrf2/HO-1 signaling pathway to enhance the antioxidant response.

**Fig. 7:**
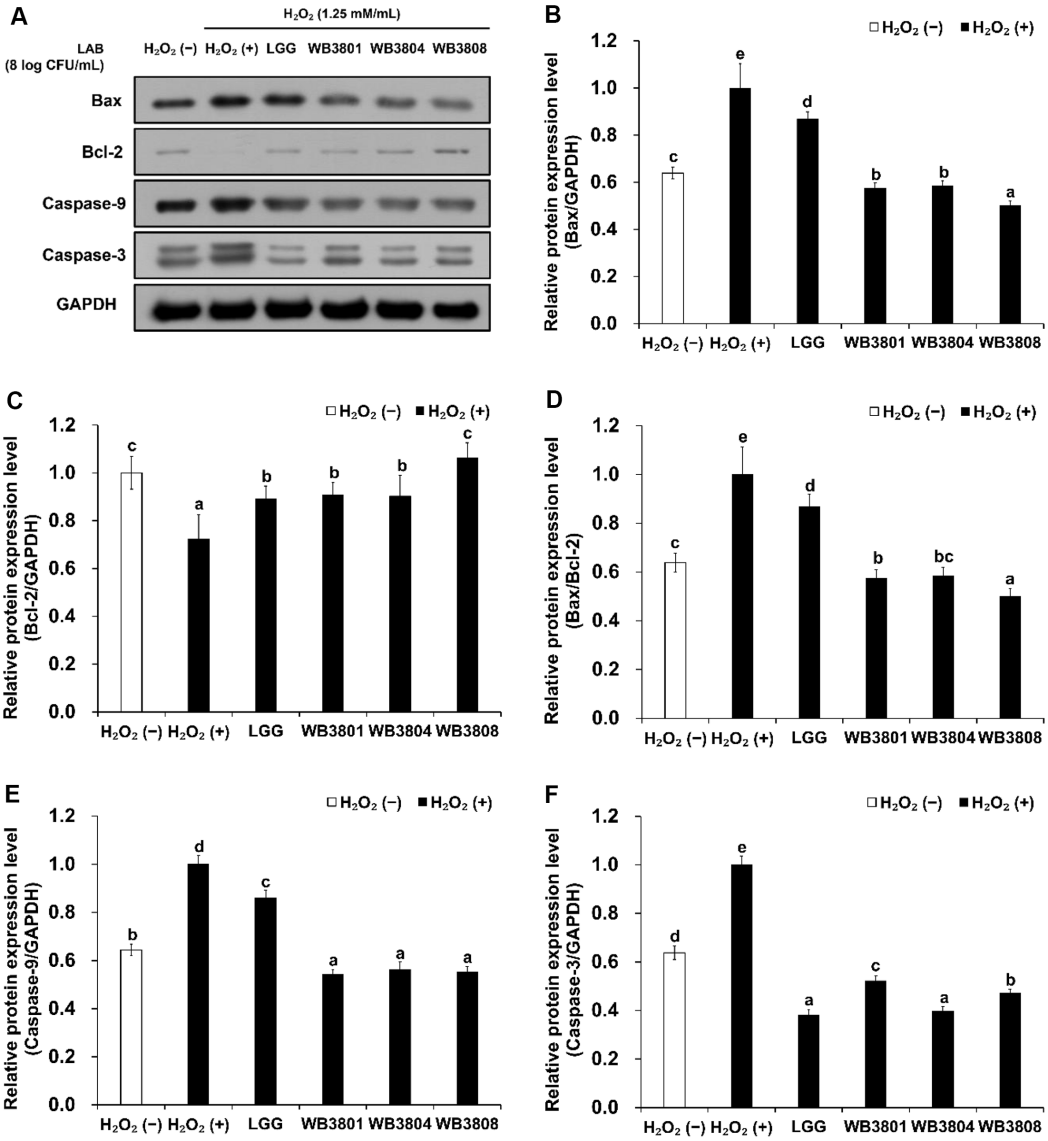
Effects of LAB strains on Bax/Bcl-2 and caspases in H_2_O_2_-induced HT-29 cells. (**A**) Western blot analysis of on apoptosis pathway, (**B**) Bax, (**C**) Bcl-2, (**D**) Bax/Bcl-2, (**E**) caspase-9, and (**F**) caspase-3. Data are presented as the mean ± SD of independent experiments. LGG, *L. rhamnosus* GG; WB3801, *L. plantarum* WB3801; WB3804, *L. plantarum* WB3804; WB3808, *L. plantarum* WB3808. ^a-d^Different letters indicate significant differences (*p* < 0.05).

In the apoptotic pathway, H_2_O_2_ stimulation elevated Bax expression and reduced Bcl-2 levels (Fig. 7), thereby increasing the Bax/Bcl-2 ratio, a key regulator of mitochondria-mediated apoptosis. Probiotics effectively downregulate the Bax/Bcl-2 ratio. Among the tested strains, WB3808 showed the most effective reduction in Bax/Bcl-2 ratio. H_2_O_2_ exposure significantly increased the expression of caspase-3 and caspase-9 compared to that in the untreated group, confirming the induction of apoptosis. All three strains elicited significant downregulation of caspase-9 expression relative to the negative control, demonstrating robust anti-apoptotic activity. In contrast, WB3804 exerted the most pronounced inhibitory effect on caspase-3 among the tested strains.

### Antioxidant Activity of LAB Strains

**Fig. 8:**
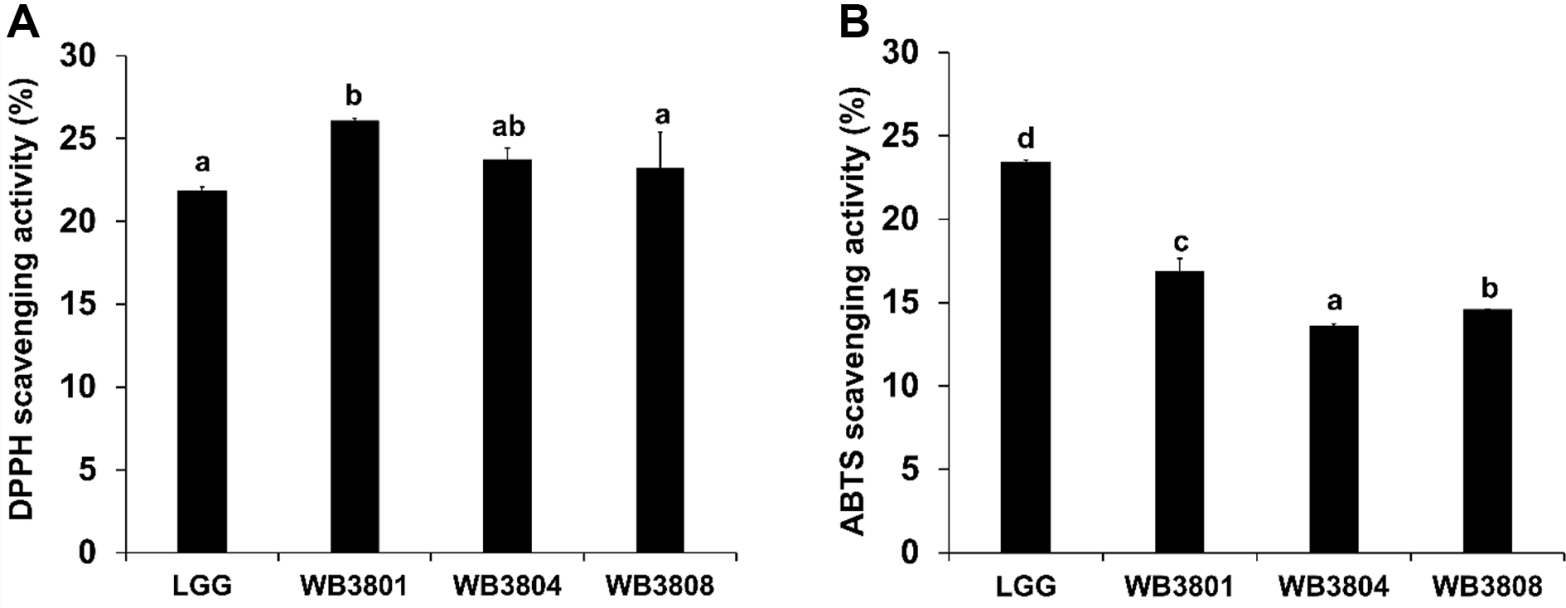
Antioxidant activities of LAB strains. (**A**) DPPH radical scavenging activity, (**B**) ABTS radical scavenging activity. Data are presented as the mean ± SD of independent experiments. LGG, *L. rhamnosus* GG; WB3801, *L. plantarum* WB3801; WB3804, *L. plantarum* WB3804; WB3808, *L. plantarum* WB3808. ^a-d^Different letters indicate significant differences (*p* < 0.05).

The antioxidant effects of the LAB strains were determined using the DPPH and ABTS assays (Fig. 8). In the DPPH assay, all probiotic groups exhibited scavenging activities exceeding 20%, and their values were higher than that of LGG (21.82%). The ABTS assay revealed that WB3801 exhibited the strongest radical-scavenging activity (16.86%), followed by WB3808 (14.58%) and WB3804 (13.59%).

## Discussion

In this study, we employed the HT-29 human colon adenocarcinoma cell line to investigate intestinal epithelial responses. However, as a cancer-derived cell line, HT-29 may not fully recapitulate the physiological characteristics of normal intestinal epithelial cells. Differences in differentiation capacity, immune receptor expression, and barrier integrity have been reported [20], which may affect the interpretation of certain responses. These limitations should be considered when analyzing the results. Despite these limitations, HT-29 remains a widely used and valuable model due to its stable growth and ability to exhibit enterocyte-like features under specific conditions [3, 5, 15].

To assess the functional probiotic capacity of L.plantarum strains, we initially screened 20 isolates from traditional Korean kimchi. WB3801, WB3804, and WB3808 were selected owing to their superior acid and bile tolerance, intestinal adherence, and cytoprotective effects compared to LGG. These strains were subjected to further analysis in H_2_O_2_-treated HT-29 cells.

Probiotic strains must resist the acidic environment of the stomach and the presence of bile salts to survive the digestive process and reach the intestinal tract in a viable form [21]. In this study, *L. plantarum* WB3801, *L. plantarum* WB3804, and *L. plantarum* WB3808 exhibited higher acid tolerance than LGG. Additionally, WB3801 and WB3808 showed superior bile salt tolerance and intestinal adhesion, indicating enhanced potential for gut colonization. These results suggest the outstanding strain-specific attributes of *L. plantarum*. The probiotic potential of *L. plantarum* is supported by its antioxidant and antimicrobial activities, gastrointestinal resistance, and intestinal adhesion capacity [22, 23]. Importantly, *L. plantarum* is regarded as safe for human consumption and has been granted the Qualified Presumption of Safety (QPS) by the European Food Safety Authority (EFSA) and is Generally Recognized as Safe (GRAS) by the United States Food and Drug Administration (FDA) [24]. The study findings further confirm that WB strains of *L. plantarum* possess robust probiotic properties, strengthening their potential as safe health-promoting candidates.

ROS production leads to oxidative stress, which results in genetic damage, protein denaturation, and lipid peroxidation. These events compromise the membrane integrity and mitochondrial function, ultimately triggering apoptotic and necrotic cell death [25]. In this study, H_2_O_2_-treated HT-29 cells were used to induce ROS production and oxidative stress-mediated apoptosis to evaluate the protective potential of WB3801, WB3804, and WB3808 against H_2_O_2_-induced oxidative toxicity. Intracellular ROS levels were assessed by fluorescence microscopy after DCFH-DA staining. DCFH-DA readily enters the cells and is enzymatically cleaved by intracellular esterases to non-fluorescent 2',7'-dichlorofluoresce, which is subsequently oxidized to highly fluorescent form in the presence of ROS [26]. Among the tested strains, WB3801 markedly reduced H_2_O_2_-induced intracellular ROS accumulation, which was consistent with its superior performance in preserving cell viability under oxidative stress conditions.

The Keap1/Nrf2/HO-1 signaling pathway plays a central cellular antioxidant defense system by regulating responses to oxidative stress. Under normal physiological conditions, Nrf2 remains retained in the cytoplasm through its interaction with Keap1, a negative regulator that promotes its ubiquitination and subsequent degradation via the proteasome pathway. Following oxidative stimuli, Nrf2 is liberated from Keap1 and translocated into the nucleus, where it activates transcription through binding to ARE sequences. This activation induces the expression of detoxifying and antioxidant enzymes, including HO-1 and NQO1 [6], which collectively reduce ROS levels and protect cells from oxidative damage [27]. In the present study, the expression of Nrf2, HO-1, and NQO1 was examined by RT-PCR and western blot analyses, indicating the activation of the Nrf2-driven antioxidant pathway. Exposure to H_2_O_2_ alone led to a modest increase in Nrf2 protein levels compared to that in the negative control, reflecting its role as a canonical oxidative stress sensor. This observation is consistent with previous reports that oxidative stimuli activate Nrf2 as part of an intrinsic cellular defense mechanism [28, 29]. Notably, WB3801 and WB3808 induced stronger activation of Nrf2 and its downstream antioxidant enzymes, resulting in potent cytoprotective effects against oxidative stress via the Nrf2/HO-1 signaling pathway. In contrast, WB3804 exhibited comparatively weaker activation of this pathway. This strain is presumed to have a lower capacity to promote the nuclear translocation of Nrf2 and the subsequent transcriptional activation of antioxidant proteins, ultimately leading to less effective suppression of intracellular ROS and apoptosis related signaling. These results highlight their potential as probiotic strains with strong antioxidant activity.

The intrinsic mitochondria-dependent apoptotic pathway plays a central role in eliminating damaged cells under oxidative stress. When ROS levels exceed the cellular antioxidant defense capacity, pro-apoptotic Bax is activated, which antagonizes anti-apoptotic Bcl-2, resulting in an increased Bax/Bcl-2 ratio. As a consequence of this imbalance, mitochondrial outer membrane integrity is compromised, allowing cytochrome c to translocate into the cytosol [30], where it initiates apoptosis via activation of the initiator caspase, caspase-9. The activated caspase-9 subsequently triggers caspase-3 as an executioner caspase, ultimately initiating an intrinsic apoptotic cascade. In this study, H_2_O_2_ exposure increased the Bax/Bcl-2 ratio and upregulated the expression of caspase-9 and caspase-3, confirming the activation of mitochondria-mediated apoptosis. Probiotics mitigate oxidative stress primarily by preserving mitochondrial membrane potential (ΔΨm), thereby preventing cytochrome c release and subsequently blocking the caspase cascade [31]. Treatment with WB3801 and WB3808 effectively reduced the Bax/Bcl-2 ratio, suppressed Bax activation, and attenuated apoptotic signaling. Flow cytometry with Annexin V/PI staining further validated these findings, showing that WB3801 and WB3808 decreased the total percentage of apoptotic cells, with WB3801 exhibiting the strongest effect.

Antioxidant enzymes such as SOD and CAT are critical for cellular protection against oxidative injury. SOD catalyses the dismutation of superoxide radicals into H_2_O_2_ and molecular oxygen, thereby initiating an enzymatic detoxification cascade. CAT decomposes H_2_O_2_ into water and molecular oxygen [32]. In this study, treatment with WB3801 and WB3808 significantly enhanced the expression of SOD and CAT. Notably, CAT protein levels showed a particularly pronounced increase in western blot analysis. These findings are consistent with those of Mu *et al*. [33]. This study demonstrates *L. plantarum* Y44 protects HT-29 cells from oxidative damage by scavenging ROS and activating intracellular antioxidant enzymes. These enzymes protect cellular components from ROS-induced oxidative damage and maintain redox homeostasis.

The direct antioxidant capacities of WB3801 and WB3808 were evaluated using DPPH and ABTS radical scavenging assays. DPPH is a stable free radical. In the presence of an antioxidant, it donates a hydrogen atom to reduce the DPPH radical. ABTS radicals are generated by reacting ABTS with potassium persulfate, and antioxidants reduce ABTS radicals, causing a decrease in color intensity [34]. Notably, WB3801 demonstrated stronger DPPH and ABTS radical scavenging activities, which is consistent with its pronounced effect on reducing intracellular ROS and enhancing antioxidant enzyme expression. These findings suggest that the cytoprotective effects of WB3801 are driven by both enzymatic antioxidant defense and direct radical scavenging, thereby alleviating oxidative stress. Furthermore, the DPPH and ABTS radical scavenging activities of food-derived LAB reported in a previous study of Kim *et al*. [35] supports our results. In this study, antioxidant activity was evaluated using DPPH and ABTS assays, which primarily reflect the electron-donating capacity of antioxidant compounds. Considering that the experimental model is based on H_2_O_2_-induced oxidative stress, incorporating a hydroxyl radical scavenging assay, as hydroxyl radical is a highly reactive species generated via the Fenton reaction, could provide a more precise assessment of the antioxidant potential of the strains.

Probiotics exhibited antioxidant effects on H_2_O_2_-induced oxidative damage model of human colon mucosal epithelial cells via Nrf2 pathway [29]. Recent studies suggest that this antioxidant response is not mediated through a single mechanism, but rather involves both secreted bioactive metabolites from microorganisms and direct physical interactions between microbes and host cells. This response is likely triggered by LAB-derived metabolites such as EPS and indole-3-lactic acid [32]. Şirin [36] found that EPS of *Lactobacillus delbrueckii* ssp. *bulgaricus* B3 and metabolites of *L. plantarum* GD2 modulates key genes and proteins within the oxidative stress related Nrf2-KEAP1 pathway. Interestingly, microbial metabolites may also facilitate or enhance host microbe contact dependent signaling, indicating that these mechanisms may act in a complementary and integrated manner. The interaction between EPS and Toll-like receptors plays a crucial role in modulating intracellular signaling cascades, leading to the activation of the Nrf2–Keap1 pathway. This activation is further facilitated by EPS–Keap1 complex formation through hydrogen bonding and electrostatic interactions, supporting its role as a potential Nrf2 activator [37]. Additionally, indole-3-lactic acid, a tryptophan metabolite of *L. plantarum* S164, alleviated intestinal barrier injury by activating the Nrf2 pathway and promoting tight junction (TJ) protein expression in an HT-29 cell model [38]. Collectively, these findings provide strong evidence that LAB-derived metabolites contribute to the activation of the Nrf2 pathway, reinforcing their role in promoting cellular antioxidant defense and intestinal barrier integrity.

Excessive ROS production and apoptosis are major drivers of intestinal barrier dysfunction and the progression of inflammatory disorders such as IBD and irritable bowel syndrome (IBS). The dual antioxidant and anti-apoptotic activities of these *L. plantarum* strains highlight their potential to protect the gut epithelium from oxidative stress–related injury and to support gastrointestinal health [39]. ROS overproduction further activates nuclear factor kappa-light-chain-enhancer of activated B cells and Mitogen-Activated Protein Kinase pathways, which increase pro-inflammatory cytokine expression and promote epithelial apoptosis [40]. Therefore, limiting ROS accumulation and maintaining the barrier integrity are essential for preserving intestinal homeostasis and preventing inflammation-induced damage. These findings suggest potential applications of functional probiotics for intestinal health and oxidative stress–related disorders.

Previous study [10] reported that *L. plantarum* ZLP001 alleviated oxidative stress in H_2_O_2_-induced IPEC-J2 intestinal epithelial cells. In comparison, the present study demonstrated that *L. plantarum* WB strains exhibited more potent cytoprotective effects under similar oxidative stress conditions. Notably, WB3801 significantly suppressed intracellular ROS levels to those comparable with untreated control and reduced apoptotic cell rates to approximately 2.51%, which is markedly lower than the 20% observed with *L. plantarum* ZLP001. These findings indicate superior antioxidant and anti-apoptotic properties. Furthermore, the WB strains showed more effective activation of the Keap1/Nrf2/HO-1 signaling pathway and inhibition of the apoptosis pathway than *L. plantarum* ZLP001. Importantly, protein-level upregulation of key antioxidant enzymes, including SOD1, CAT, and HO-1, was confirmed via Western blot analysis in the WB strains, a feature not addressed in the *L. plantarum* ZLP001 study. These results suggest that the WB strains possess a more robust and multifaceted antioxidant mechanism, supporting their potential as functional probiotics for maintaining intestinal epithelial health. Although this study demonstrated antioxidant and anti-apoptotic effects of *L. plantarum* WB3801 and WB3808 in an *in vitro* HT-29 cell model, further validation is needed to evaluate their survivability, colonization ability, and bioavailability following oral administration. Future *in vivo* studies and clinical trials will be essential to confirm the therapeutic potential of these strains.

## Conclusion

*L. plantarum* strains (WB3801, WB3804, and WB3808) exerted significant cytoprotective effects against H_2_O_2_-induced oxidative stress in HT-29 cells. These strains enhance cellular antioxidant defenses by activating the Keap1/Nrf2/HO-1 signaling pathway, upregulating antioxidant enzymes including SOD1 and CAT, and exhibiting notable free radical scavenging activity. Furthermore, treatment with these strains effectively attenuated oxidative stress–mediated apoptosis by reducing the Bax/Bcl-2 ratio and suppressing the activation of caspase-9 and caspase-3. Collectively, these findings suggest that *L. plantarum* strains, particularly WB3801 and WB3808, possess strong probiotic potential for maintaining intestinal homeostasis and protecting against oxidative stress–related epithelial injury. Further *in vivo* studies are necessary to validate their functional effectiveness and explore their potential utility in preventing intestinal disorders.

## Figures and Tables

**Table 1:** Primers utilized for qRT- PCR.

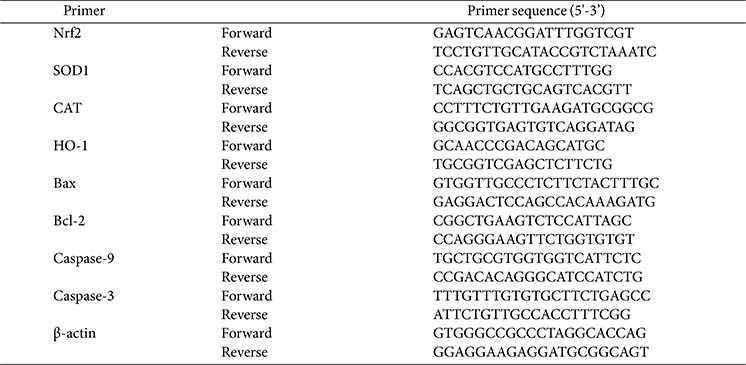

**Table 2:** Measurement of artificial gastric conditions tolerance and adhesion ability of LAB strains.